# Epigenetic effects of high-fat diet on intestinal tumorigenesis in C57BL/6J-*Apc*^*Min*/+^ mice

**DOI:** 10.20517/jtgg.2022.16

**Published:** 2023-01-31

**Authors:** Dan C. Qu, Devin Neu, Zain Q. Khawaja, Ruoyu Wang, Cynthia F. Bartels, Katreya Lovrenert, Ernest R. Chan, Anne E. Hill-Baskin, Peter C. Scacheri, Nathan A. Berger

**Affiliations:** 1 Center for Science, Health and Society, Case Western Reserve University School of Medicine, Cleveland, OH 44106, USA.; 2 Department of Genetics and Genome Sciences, Case Western Reserve University School of Medicine, Cleveland, OH 44106, USA.; 3 Cleveland Institute for Computational Biology, Case Western Reserve University, Cleveland, OH 44106, USA.; 4 Case Comprehensive Cancer Center, Case Western Reserve University, Cleveland, OH 44106, USA.

**Keywords:** Obesogenic diet, epigenetic changes, lipid metabolic processes, *Apc* ^*Min*/+^

## Abstract

**Aim:**

Obesity and obesogenic diets might partly accelerate cancer development through epigenetic mechanisms. To determine these early effects, we investigated the impact of three days of a high-fat diet on epigenomic and transcriptomic changes in *Apc*^*Min*/+^ murine intestinal epithelia.

**Method:**

ChIP-Seq and RNA-Seq were performed on small intestinal epithelia of WT and *Apc*^*Min*/+^ male mice fed high-fat diet (HFD) or low-fat diet (LFD) for three days to identify genomic regions associated with differential H3K27ac levels as a marker of variant enhancer loci (VELs) as well as differentially expressed genes (DEGs).

**Results:**

Regarding epigenetic and transcriptomic changes, diet type (LFD *vs*. HFD) showed a significant impact, and genotype (WT *vs*.*Apc*^*Min*/+^) showed a small impact. Compared to LFD, HFD resulted in 1306 gained VELs, 230 lost VELs, 133 upregulated genes, and 127 downregulated genes in WT mice, with 1056 gained VELs, 371 lost VELs, 222 upregulated genes, and 182 downregulated genes in *Apc*^*Min*/+^ mice. Compared to the WT genotype, the *Apc*^*Min*/+^ genotype resulted in zero changed VELs for either diet type group, 21 DEGs for LFD, and 48 DEGs for HFD. Most gained VELs, and upregulated genes were associated with lipid metabolic processes. Gained VELs were also associated with Wnt signaling. Downregulated genes were associated with antigen presentation and processing.

**Conclusion:**

Three days of HFD-induced epigenomic and transcriptomic changes involving metabolic and immunologic pathways that may promote tumor growth in the genetically predisposed murine intestine without affecting key cancer signaling pathways.

## INTRODUCTION

Colorectal cancer is an obesity-associated cancer where excess body fat promotes cancer development and worsens outcomes in patients with these tumors^[1]^. Using mouse models, we and others have shown that obesogenic diets and obesity accelerate the development of intestinal neoplasia in C57 BL6J mice with mutation or deletion of the APC gene^[2,3]^. Obesity has been postulated to accelerate cancer development partly through epigenetic mechanisms^[4]^. Murine models in which mice were fed a high-fat diet (HFD) for at least 15–20 weeks have, in fact, shown changes in histone H3 acetylation and DNA methylation associated with remodeling of chromatin regulatory regions that resemble cancer progression^[5,6]^. However, we have previously shown appearances of intestinal polyps in*Apc*^*Min*/+^ mice within three days of starting on high-fat obesogenic diets, even prior to excessive weight gain^[2]^. Accordingly, we used ChIP-Seq, and RNA-Seq approaches to investigate if a three-day HFD was sufficient to induce epigenetic or transcriptional changes supportive of tumor growth in intestinal epithelia of*Apc*^*Min*/+^ and C57 BL6J mice. ChIP-Seq analysis focused on identifying VEL and the proximal genes they may regulate. In mammalian cells, active enhancer elements that regulate transcriptional promoters by *cis*-acting mechanisms are located within open chromatin and are characterized by epigenetic alterations in histone methylation and acetylation. Enhancers have been shown to vary under multiple conditions, including stages of cell differentiation and malignant transformation. These regions, marked with high levels of H3K4me1 and H3K27ac, have been termed variant enhancer loci (VELs) and are associated with transcriptional changes of crucial cancer genes in human colon cancer lines and human colorectal cancer tumors^[7,8]^.

## METHODS

### Mice

C57BL/6J (denoted as *Apc*^+/+^ or wild-type) and C57BL/6J-*Apc*^*Min*/+^ (denoted as*Apc*^*Min*/+^) mice were purchased from the Jackson Laboratory (Bar Harbor, ME) to develop breeding colonies that supplied all mice used in the study. Mice were maintained in micro isolator cages at 25 °C on a 12 h reverse light/dark cycle at the Case Western Reserve University (CWRU) Animal Resource Facility. Breeding was accomplished by placing one adult mutant (*Apc*^*Min*/+^) male mouse with two WT adult female *Apc*^+/+^ mice to generate mutant and WT littermates, which were subsequently distributed to experimental diets as previously described^[2]^. Ear punch samples were taken from mice at 14 days of age for genotyping. Offspring were weaned and separated by sex at 21 days of age. Siblings were incubated together and fed a standard chow diet Prolab RMH 3000 (5P00; LabDiet; Brentwood, MO) with autoclaved water *ad libitum*. At 30 days of age, sibling male mice were separated by genotype, wild-type or*Apc*^*Min*/+^, distributed to an experimental diet (high-fat or low-fat) with autoclaved water, and then incubated in groups of two or three mice per cage until sacrifice after three days on experimental diet.

### Experimental diets

Experimental diets consisted of a high-fat diet (HFD) and a low-fat diet (LFD), as previously described^[2,9]^. These diets differed in amounts of fats from hydrogenated coconut oil. The HFD contained 58.0% kcal/g of fat, 25.5% kcal/g of carbohydrate, and 16.4% kcal/g of protein (D12330; Research Diets; New Brunswick, NJ). The LFD contained 10.5% kcal/g of fat, 73.1% kcal/g of carbohydrate, and 16.4% kcal/g of protein (D12328; Research Diets; New Brunswick, NJ). Diets were otherwise matched for all micronutrients. At 30 days of age, male WT and*Apc*^*Min*/+^ mice were assigned to either the HFD or LFD, resulting in four groups according to genotype and diet: WT-LFD, WT HFD,*Apc*^*Min*/+^-LFD,*Apc*^*Min*/+^-HFD. Mice were fed experimental diets *ad libitum* for three days (30–33 days of age). Biological replicate mice were used for each combination of genotype and diet type. All animal procedures were evaluated and approved by the Institutional Animal Care and Use Committee of CWRU School of Medicine, Protocol Number 2020-016.

### Intestinal epithelia isolation

After three days on experimental diets, mice were euthanized by cervical dislocation. Small intestines were immediately removed, flushed with ice-chilled phosphate-buffered saline (PBS), and cut longitudinally. Mesenteric tissue was removed from the intestines. Each small intestine was then cut transversely into four equally sized strips. The intestinal strips were gently agitated in EDTA-based Cell Dissociation Buffer (13151014; Thermo Fisher Scientific; Waltham, MA) at 4 °C for 45 min, rinsed in cold PBS, and then agitated again in fresh dissociation buffer at 4 °C for another 45 min. Tissue strips were cut into small pieces, approximately 2 mm × 2 mm. Tissue pieces were rapidly pipetted up and down in cell dissociation buffer with a 10 mL serological pipet for 5 min or until the solution became cloudy. Intestinal pieces were removed from the solution using a 150 μm sterile paint straining mesh (2650; Mutual Dropcloth Company; Monroe, NC). The suspension was centrifuged at 200× *g* for 5 min, and the supernatant was aspirated, leaving a pellet of intestinal epithelia. The pellets were then suspended in 10.5 mL of cold PBS in preparation for ChIP-Seq (10 mL) and RNA-Seq (500 μL). The samples were immediately flash frozen with liquid nitrogen and stored at −80 °C for later use.

### ChIP-Seq

ChIP-Seq was performed on each 10 mL intestinal epithelia sample as previously described^[10]^. The Covaris truChIP^™^ Chromatin Shearing Kit (520154; Covaris; Woburn, MA) was used to cross-link the intestinal epithelia and extract their cell nuclei according to the manufacturer’s protocol. Samples were sheared using the Covaris model S2 AFA focused ultrasonicator with the following settings: duty cycle-5%, intensity-4, cycles/burst-200, time-seven minutes). Chromatin immunoprecipitation using 9 μg of rabbit anti-H3K27ac antibody (ab4729; Abcam; Cambridge, UK) followed by recovery of DNA and then preparation of ChIP-Seq libraries were performed as previously described^[11]^. ChIP-Seq libraries were sequenced on an Illumina NextSeq 550 system (Illumina; San Diego, CA) at the CWRU Genomics Core.

The FASTX-Toolkit v0.013 was used to remove adapter sequences and trim read ends using a quality score cutoff of 20 (Available from: http://hannonlab.cshl.edu/fastx_toolkit/). ChIP-seq data were aligned to the mm9 genome assembly using Bowtie 2 v2.3.4.3, discarding reads with at least one mismatch and reporting the best alignment if multiple alignments were present^[12]^. Output SAM files were converted to binary (BAM) format, sorted, indexed, and PCR duplicates were removed using SAMtools v1.10^[13]^. Peaks were detected using MACS2 v2.1.2 with default settings and the broad flag set^[14]^. Peak lists were filtered to remove all peaks overlapping ENCODE blacklisted regions^[15,16]^. DeepTools v3.2.0 was used to generate RPGC-normalized bigWig tracks with 50 bp bin sizes of the final sample BAM files^[17]^. bigWig tracks were visualized on the Integrative Genomics Viewer to assess samples for any track irregularities or low signal-to-noise ratio^[18]^.

### Identification of variant enhancer loci

H3K27ac peaks called across samples were filtered for significance (Benjamini-Hochberg corrected *P* ≤ 0.001). Overlapping peaks were merged, and the read depth for each peak region across samples was determined using BEDTools v2.17.0, generating a count matrix^[19]^. DESeq2 v1.34, a tool used to analyze many kinds of high-throughput count data, including ChIP-Seq, was used to identify regions with peaks that have significant differential enrichment of H3k27ac activity (Benjamini-Hochberg corrected *P* ≤ 0.05) relative between the four different groups of mice: WT-LFD, WT HFD,*Apc*^*Min*/+^-LFD,*Apc*^*Min*/+^-HFD^[20]^. The effect size estimate for the differential enrichment between the two groups at a specific H3K27ac region was measured as log_2_(fold-change). These regions of differential enrichment were termed VEL^[7]^.

### RNA-Seq

RNA was isolated from the intestinal epithelia samples using 1 mL of TRIzol (Life Technologies; Carlsbad, California; 15596-026) according to the manufacturer’s protocol by the CWRU Translational Resource Core. RNA-seq libraries were prepared and sequenced on an Illumina NextSeq 550 platform by the CWRU Genomics Core. Data were assessed for quality and trimmed for adapter sequences using Trim Galore! v0.4.2 (Babraham Bioinformatics; Cambridge, UK), a wrapper script for FastQC and Cutadapt. Reads that passed quality control were then aligned to the mouse reference genome (mm9) using the STAR aligner v2.5.3^[21]^. Alignment for the sequences was guided using the GENCODE annotation for mm9. Reads were analyzed for differential expression using Cufflinks v2.2.1, an RNA-Seq analysis package that reports the fragments per kilobase of exon per million fragments mapped for each gene^[22]^. A differential analysis report was generated using the *cuffdiff* command performed pairwise for each mouse group to identify differentially expressed genes (DEGs). The effect size estimate of each gene’s differential expression between the two groups was measured as log_2_(fold-change). Significant differential expression was identified using a cutoff of *q*-value < 0.05.

### Functional enrichment analysis

Using the default regulatory domain definition (basal promoter 5 + 1 kb and extension up to 1 Mb), Genomic Regions Enrichment of Annotations Tool (GREAT) v4.04 was used to identify genes predicted to be associated with ChIP-Seq VELs^[23]^. Search Tool for the Retrieval of Interacting Genes/Proteins (STRING) v11.5 was then used to identify biological pathways from the KEGG pathway database found to be significantly associated with these predicted genes (Benjamini-Hochberg corrected *P* ≤ 0.05)^[24]^. STRING was also used to identify pathways significantly associated with DEGs identified from RNA-Seq.

### Correlation of VELs and target gene expression

DEGs predicted to be associated with VELs were identified according to the analysis of both ChIP-Seq and RNA-Seq data. The list of DEGs found from RNA-Seq was compared to the list of genes predicted by GREAT to be associated with the VELs found from ChIP-Seq. For each identical gene found in both lists, the RNA fold-change of the DEG was compared to the average H3K27ac fold-change of all of the associated VELs. VEL-gene associations in which both fold-changes were concordant (increase in RNA fold-change and increase in H3K27ac fold-change OR decrease in RNA fold-change and decrease in H3K27ac fold-change) were identified.

### Hypergeometric optimization of enrichment analysis

Hypergeometric optimization of enrichment (HOMER) software was used to identify *de novo* motifs among the VELs (Available from: http://homer.ucsd.edu/homer/)^[25]^. These *de novo* motifs were then compared to known motifs to find the closest match to identify putative transcription factors that may be involved in interaction with the VELs.

## RESULTS

### High-fat diet-induced epigenomic and transcriptomic changes

**Figure 1.**
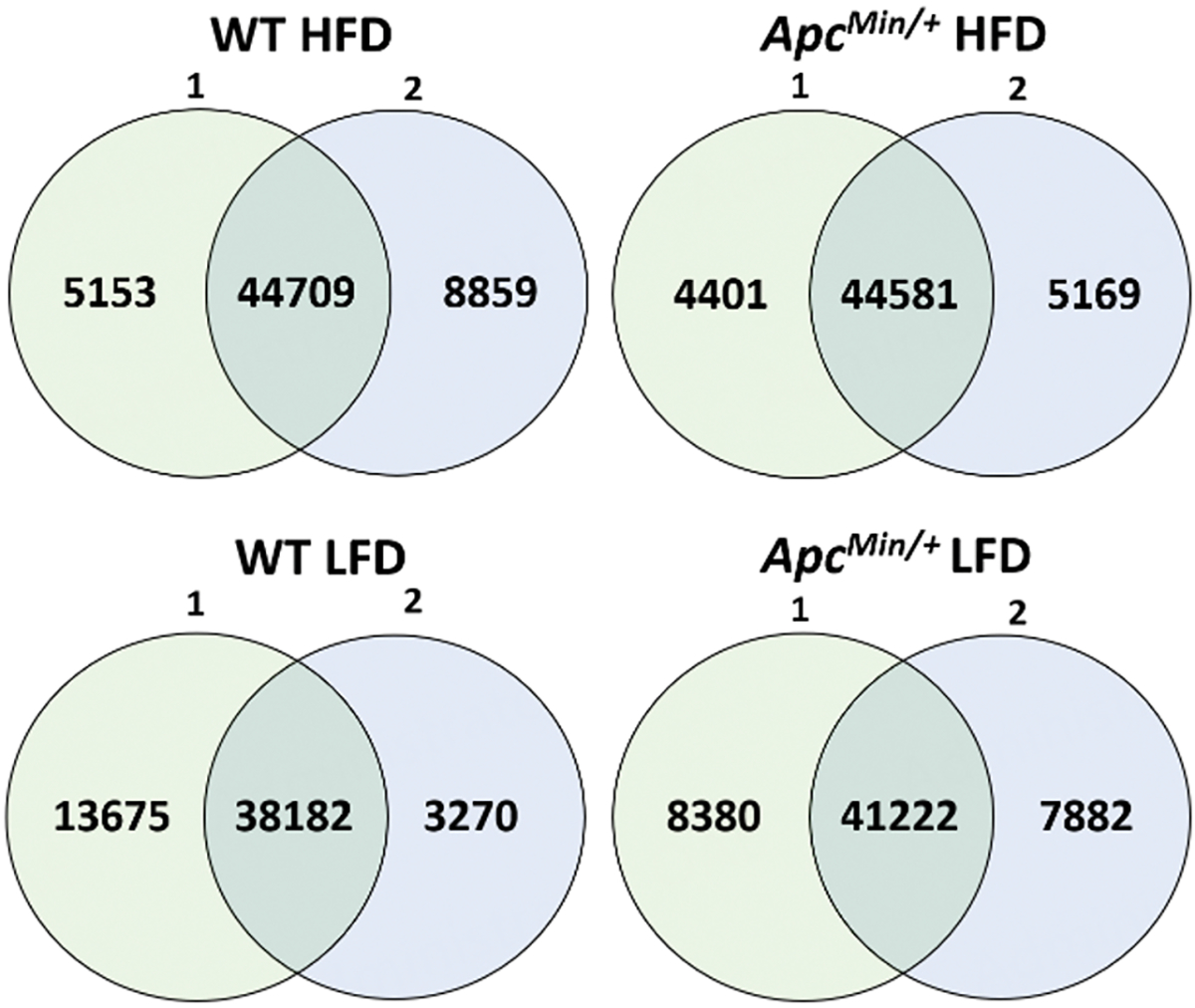
The number of H3K27ac peaks overlapping between the biological replicates as well as the number of peaks exclusive to each replicate for each mouse group.

H3K27ac ChIP-Seq profiles were obtained from the small intestinal epithelia samples of WT and*Apc*^*Min*/+^ mice fed with either HFD or LFD for three days. More than 40,000 H3K27ac peaks were identified for each sample. Comparison between biological duplicates showed mostly overlapping H3K27ac peaks [Figure 1].

**Figure 2.**
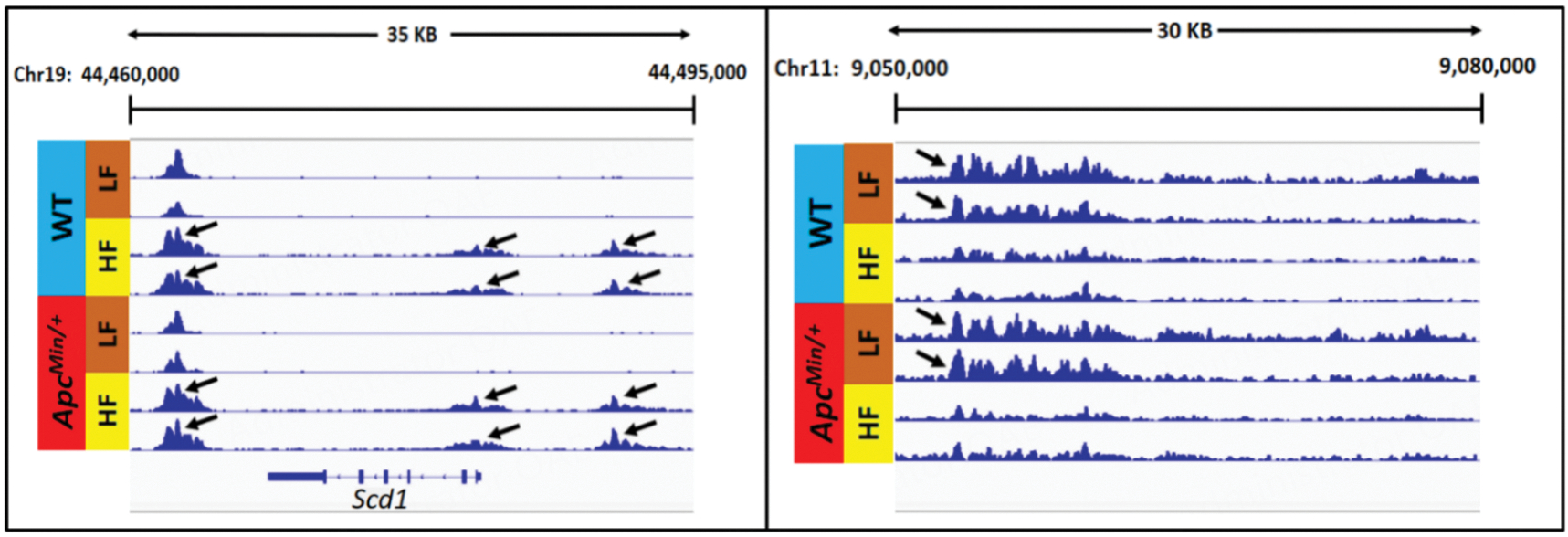
Normalized ChiP-Seq tracks illustrating representative examples of gained VELs (left panel) and lost VELs (right panel) induced by HF diet.

A representative browser view of the H3K27ac ChIP-Seq data signal is shown in Figure 2. Gained VELs were defined as VELs with increased H3K27ac enrichment when comparing HFD profiles to LFD profiles. Lost VELs were defined as VELs with a decrease in H3K27ac enrichment when comparing HFD profiles to LFD profiles.

**Figure 3.**
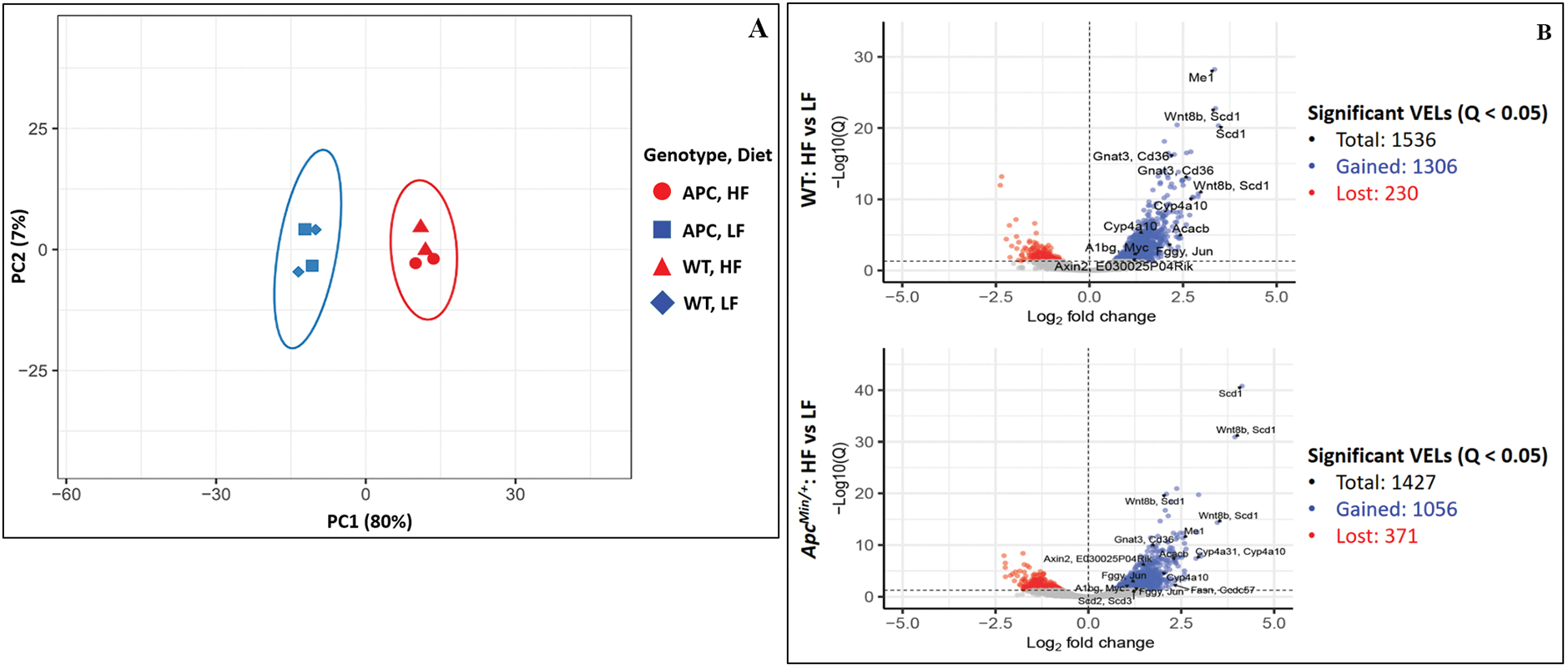
(A) Principal component analysis of H3K27ac profiles of the small intestinal crypt samples. (B) Total number of significant VELs identified in comparison between HFD and LFD (Bonferroni corrected *P* < 0.05) with certain notable VELs labeled with nearby genes. The corresponding data for these labeled VELs are shown in Supplementary Figure 6.

Principal component analysis showed that the H3K27ac profiles of HFD samples were highly distinct from those of LFD samples [Figure 3A]. Using BEDTools, the H3K27ac peak profiles of biological replicates were merged: 93,309 peaks were identified for WT LFD, 103,430 for WT HFD, 98,706 for*Apc*^*Min*/+^ LFD, and 98,732 for*Apc*^*Min*/+^ HFD. When directly comparing the merged H3K27ac profile of HFD samples to that of LFD samples for each genotype using DESeq2, a total of 45,910 VELs were identified for WT mice, 1536 of which were statistically significant (3.35%), and 48,646 VELs were identified for*Apc*^*Min*/+^ mice, 1427 of which were statistically significant (2.93%) [Figure 3B]. For WT mice, the log_2_(fold-change) of 967 gained VELs was > 1, and 173 lost VELs were < −1. For*Apc*^*Min*/+^ mice, the log_2_(fold-change) of 824 gained VELs were > 1, and of 270 lost VELs were < −1 [Figure 3B]. In contrast to the impact of diet type, the epigenetic impact of genotype was not as significant. According to principal component analysis, only a small variance was demonstrated between WT HFD samples and*Apc*^*Min*/+^ HFD samples [Figure 3A]. Additionally, DESeq2 did not identify any significant VELs when comparing WT HFD profiles to*Apc*^*Min*/+^ HFD profiles or when comparing WT LFD profiles to*Apc*^*Min*/+^ LFD profiles. Overall, HFD, in only three days, was able to cause epigenetic changes within the intestinal epithelia.

GREAT found multiple genes predicted to be associated with the identified VELs. In WT mice, 1660 genes were predicted to be associated with gained VELs, and 345 genes were predicted to be associated with lost VELs; In*Apc*^*Min*/+^ mice, 1434 genes were predicted to be associated with gained VELs, and 590 genes were predicted to be associated with lost VELs. The distributions of the VELs relative to the transcription start site (TSS) of annotated genes were relatively similar regardless of genotype. More than 90% of the VELs were localized more than 5 kb away from the nearest TSS, suggesting that they predominantly function as enhancers.

**Figure 4.**
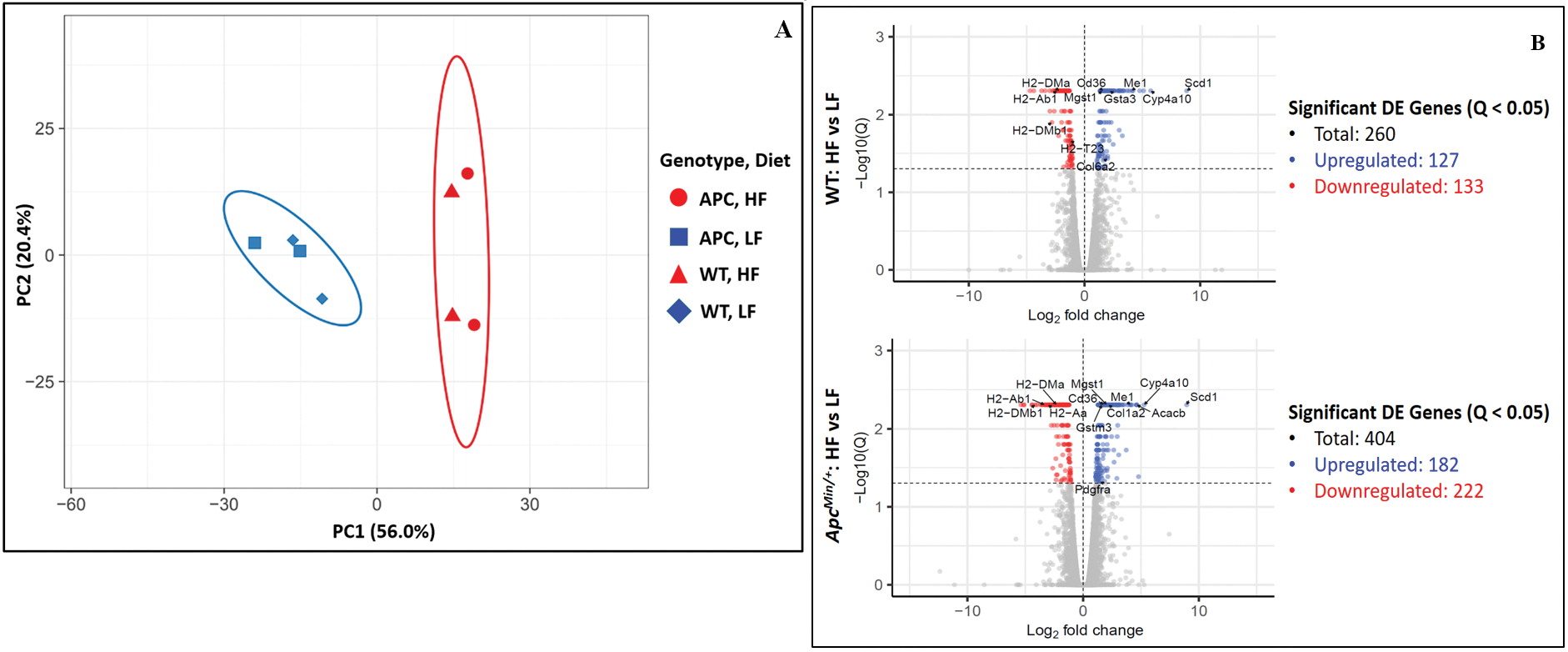
(A) Principal component analysis of RNA expression profiles of the small intestinal crypt samples. (B) Total number of DEGs identified in comparison between HFD to LFD (FDR *Q*-value < 0.05) with certain notable DEGs labeled. The corresponding data for these labeled DEGs are shown in Supplementary Figure 7.

RNA samples of adequate quality were isolated from the same epithelial samples used to obtain ChIP-Seq epigenomic profiles to generate RNA-Seq transcriptomic profiles [Supplementary Figure 1]. Similar to the epigenomic profiles, principal component analysis of the transcriptomic profiles showed a significant distinction between HFD samples and LFD samples and a less obvious distinction between similar WT samples and*Apc*^*Min*/+^ samples when controlling for diet type [Figure 4A]. The transcriptomic profiles of the different sample types were directly compared to identify all DEGs. Many DEGs were identified when directly comparing HFD samples to LFD samples [Figure 4B]. Relatively few DEGs were identified when directly comparing*Apc*^*Min*/+^ samples to WT samples while controlling for diet [Supplementary Figure 2]. Overall, the RNA-Seq results were similar to the ChIP-Seq results in that diet type had a much more significant overall impact than differences in genotype.

### Impact of HFD on lipid metabolism supported by epigenomic and transcriptomic changes

**Figure 5.**
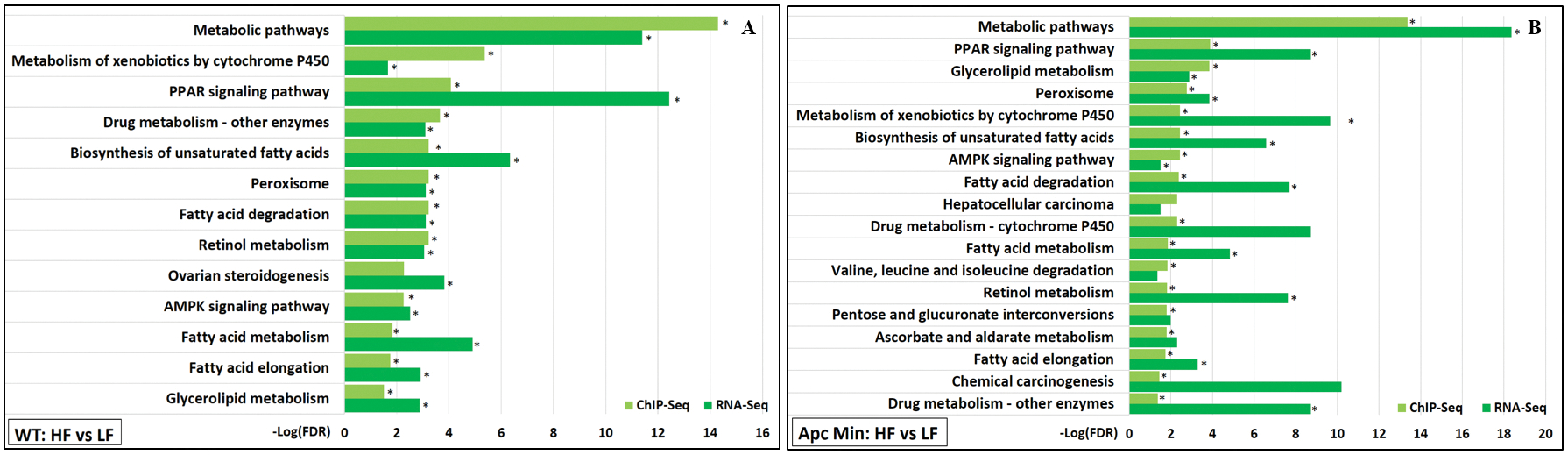
KEGG pathway functional enrichments significantly associated with both gained VELs (ChIP-Seq) and upregulated genes (RNA-Seq) induced by high-fat diet were identified for (A) WT mice and (B)*Apc*^*Min*/+^ mice. An asterisk next to a light green bar indicates that this pathway is identified to be significantly associated with gained VELs in both genotypes. An asterisk next to a dark green bar indicates that this pathway is identified to be significantly associated with upregulated genes in both genotypes.

Multiple KEGG pathway enrichments were identified from HFD-induced gained VELs revealed by ChIP-Seq and HFD-induced upregulated genes revealed by RNA-Seq. Most of these pathways and processes were involved in lipid metabolism, including the “PPAR signaling pathway”, “AMPK signaling pathway”, “biosynthesis of unsaturated fatty acids”, “fatty acid elongation”, and “fatty acid degradation” [Figure 5].

Although GREAT analysis of the ChIP-Seq data predicted that nearly 2000 genes were associated with gained VELs induced by HFD for each genotype, only a fraction of those genes were found to be differentially expressed according to RNA-Seq when comparing the HFD samples to the LFD samples. From analyses of the ChIP-Seq and RNA-Seq results, 76 genes upregulated in WT mice and 99 genes upregulated in*Apc*_*Min*/+_ mice were also predicted to be associated with gained H3K27ac VELs [Supplementary Figure 3A]. Similar analysis revealed 39 genes downregulated in WT mice and 40 genes downregulated in A*pc*_*Min*/+_ were predicted to be associated with lost H3K27ac VELs. [Supplemental Figure 3B]

Most upregulated genes predicted to be associated with gained H3K27ac were shown to be involved in the previously mentioned lipid metabolic processes and pathways. Notably, upregulated genes included those involved in fatty acid syntheses such as fatty acid synthase (*Fasn*), stearoyl-CoA desaturases (*Scd1*, *Scd2*), acetyl-CoA carboxylase 2 (*Acacb*), and NADP-dependent malic enzyme (*Me1*); fatty acid oxidation such as cytochrome P450 A10 (*Cyp4a10*) and acetyl-Coenzyme A acyltransferase 2 (*Acaa2*); and fatty acid transport such as fatty acid translocase (*Cd36*). Overall, the ChIP-Seq and RNA-Seq analyses show evidence that the upregulation of genes involved in lipid metabolism by HFD may have a critical epigenetic mechanism.

### Many epigenomic changes associated with wnt signaling

**Figure 6.**
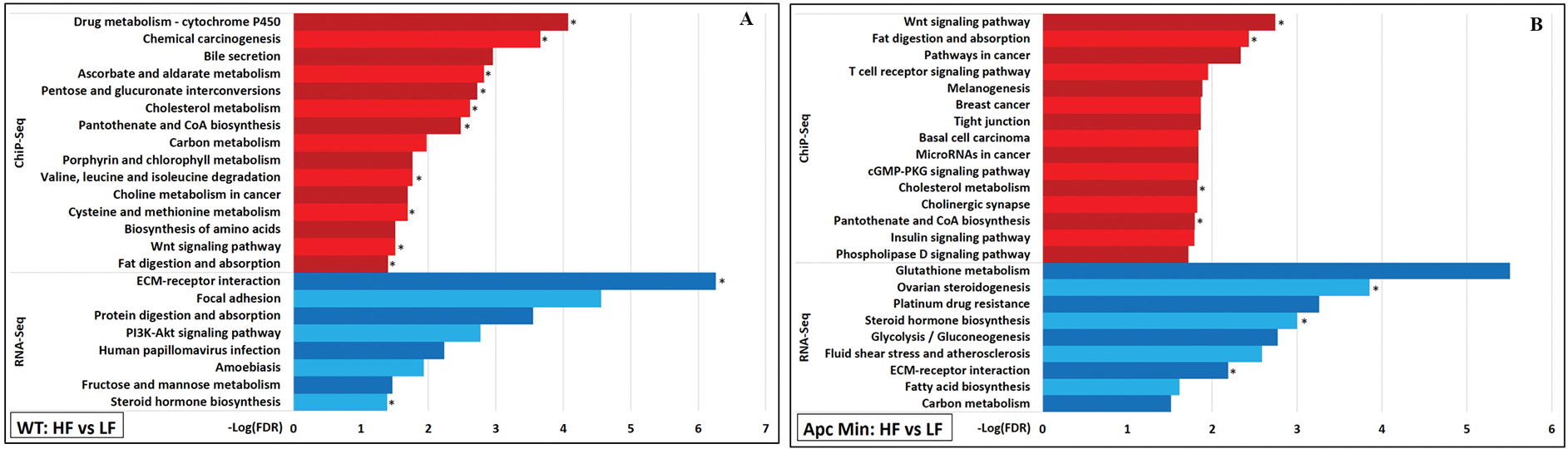
For (A) WT mice and for (B)*Apc*^*Min*/+^ mice, the top 15 most significant KEGG pathways associated with only gained VELs (ChIP-Seq) and not with upregulated genes (RNA-SEQ) were denoted with red bars, and top 15 most significant pathways associated with only upregulated genes and not with gained VELs were denoted with blue bars. An asterisk next to a bar indicates that this pathway is significantly associated with gained VELs (red bar) or upregulated genes (blue bar) in both genotypes.

Several HFD-induced gained VELs were predicted to be associated with genes involved in the “Wnt signaling pathway”, with 41 VELs found in WT mice and 43 in*Apc*^*Min*/+^ mice [Figure 6 and Supplementary Figure 4]. Some of these commonly gained VELs include those predicted to be associated with genes that encode Wnt ligands (*Wnt3*, *Wnt8b*), the negative Wnt regulator *Axin2*, and downstream target proto-oncogenes *Myc* and *Jun*. The Wnt pathway has been implicated as one of the signaling networks important for coordinating metabolic status with various biological processes, including cell proliferation, growth arrest, differentiation, and apoptosis^[26]^. Interestingly, additional functional enrichments related to different cancers that also involve Wnt signaling (“hepatocellular carcinoma”, “breast cancer”, “basal cell carcinoma”) were identified from the genes predicted to be associated with the gained VELs in*Apc*^*Min*/+^ mice [Figure 6B]^[27]^. Despite these epigenetic changes, none of these genes involved in Wnt signaling were identified to be upregulated by RNA-Seq [Figure 6]. Interestingly, a previous study that fed HFD to mice for 15–20 weeks also resulted in epigenomic changes associated with Wnt signaling without notable transcriptomic changes^[5]^.

Aside from Wnt signaling, HFD was noted to upregulate the transcription of two small distinct sets of genes related to cancer. In WT mice samples, the “PI3K-Akt signaling pathway” was identified as being significantly enriched because genes encoding ligands (*Col1a1*, *Col1a2*, *Col4a1*, *Col6a2*, *Col6a3, Lamb3*, *Tnc*) and receptors (*Pdgfrb*, *Pdgfra*) that make up the “ECM-receptor interaction” and function in “focal adhesion” utilized this signaling pathway [Figure 6A]^[27]^. Aside from *Lamb3,* these genes were not identified as being associated with any VELs identified from ChIP-Seq. In*Apc*^*Min*/+^ samples, HFD upregulated enzymes involved in glutathione metabolism, which is important for controlling oxidative stress and resistance to chemotherapeutic agents [Figure 6B]^[28,29]^. These genes include *Gstm3*, *Gstm1*, *Mgst1*, *Gsta3*, *Gstm4*, *Mgst2*, and *Gm10639*. Aside from *Mgst1*, these genes were not identified as associated with VELs.

### HFD downregulated many genes without associated H3K27ac changes

**Figure 7.**
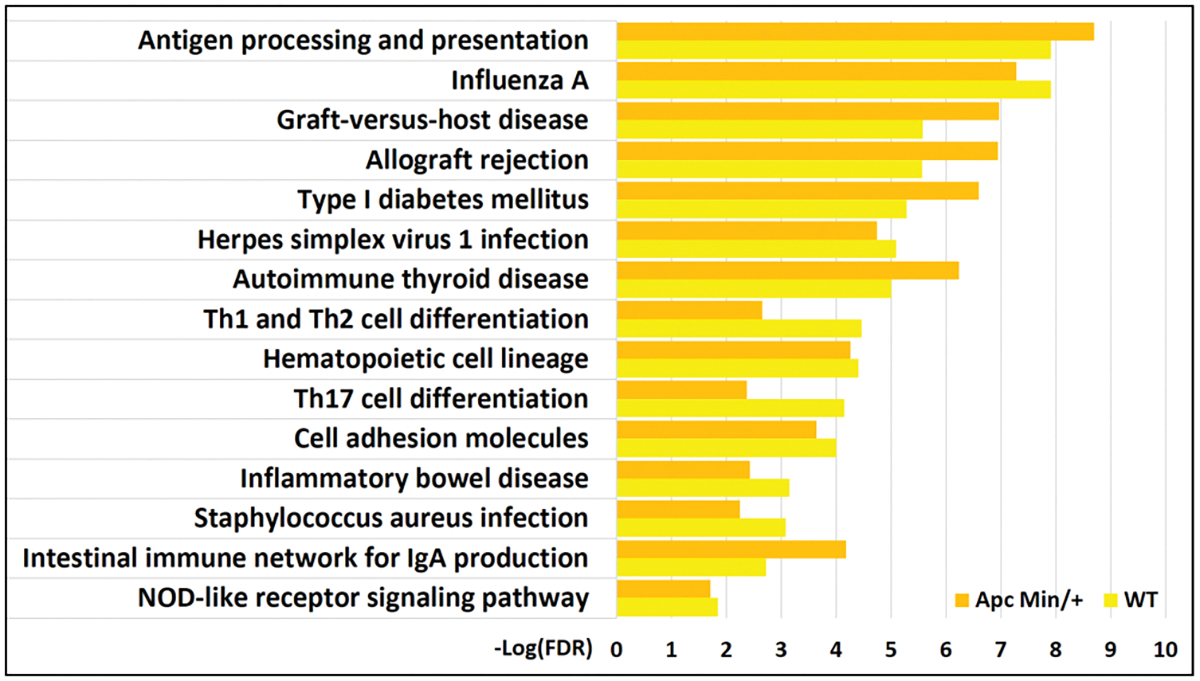
KEGG pathways significantly associated with genes downregulated by high-fat diet (RNA-Seq) in both WT and*Apc*^*Min*/+^ mice.

Functional enrichment analysis of the RNA-Seq showed that several genes downregulated by HFD were associated with processes involved in the immune system [Figure 7]. There did not appear to be any lost H3K27ac correlated with the downregulation of these processes. In fact, unlike for gained VELs, no KEGG pathway enrichments were identified for lost VELs except for the “phosphatidylinositol signaling system.” The most notable immune process identified to be downregulated “antigen processing and presentation.” This process is also crucial in differentiating T cells such as Th1, Th2, and Th17^[27]^. Notable genes encode MHC II proteins such as *H2-DMa*, *H2-Dmb1*, and *H2-Ab1*; MHC I proteins such as *H2-T23* and *Gm11127*; and the transcriptional coactivator *Ciita* involved in the transcription of MHC proteins.

Previous studies have shown that NOD-like receptors, intracellular sensors part of the innate immune response that detect compounds associated with pathogens and cell stress, are involved in MHC class II expression^[30]^. Genes involved in the “NOD-like receptor signaling pathway” were also found to be downregulated by an HFD in our study in WT mice (*Cyba*, *Nfkbia*, *Casp4*, *Oas3*, *Ccl5*, *Il18*) and*Apc*^*Min*/+^ mice (*Irf7*, *Casp4*, *Oas3*, *Ccl5*, *Gbp7*, *Oas2*, *Oas1a*) [Figure 7]. Pro-inflammatory cytokine interferon-gamma signaling has previously been shown to downregulate MHC class II expression^[30]^. STRING identified genes associated with the GO Biological Process term “response to interferon-gamma” in WT mice (*Nos2, Ciita, Ubd, Ccl5, H2-Ab1, Ccl20*) and*Apc*^*Min*/+^ mice (*Ccl24, Nos2, Ciita, Ubd, Ccl5, H2-Ab1, H2-Aa, Nmi*). N-myc-interactor (*Nmi*) interacts with STATs in the JAK-STAT pathway to augment STAT-mediated transcription in response to cytokines IL-2 and IFN-gamma. Besides MHC class II genes, other downstream target genes are transcribed by STATs. Nitric oxide synthase 2 (*Nos2*) produces nitric oxide, a reactive free radical that mediates antimicrobial and antitumor activities. Ubiquitin D (*Ubd*) may be involved in dendritic cell maturation and the regulation of TNF-alpha-induced and LPS-mediated activation of NF-kappa-B, the central mediator of innate immunity. CC chemokines (*Ccl5*, *Ccl24*) induce chemotaxis in specific leukocytes.

### Predicted transcription factors associated with VELs were identified

HOMER analysis identified six likely *de novo* DNA motifs for WT HF *vs*. LF gained VELs, five for*Apc*^*Min*/+^ HF *vs*. LF gained VELs, one for WT HF *vs*. LF lost VELs, and two for*Apc*^*Min*/+^ lost VELs [Supplementary Figure 5]. One of the *de novo* motifs from the WT gained VELs matched similarly to the known binding motif for Fos, a proto-oncogenic transcription factor part of the AP-1 complex involved in cell proliferation, differentiation, and transformation. One of the *de novo* motifs from the*Apc*^*Min*/+^ gained VELs closely matched with the known binding motif for PPAR-α, which upregulates genes involved in the transport, binding, activation, and oxidation of fatty acids. Another *de novo motif* from the*Apc*^*Min*/+^ gained VELs closely matched with a known binding motif for Jun-B, another component of the AP-1 complex. The one *de novo* motif identified for WT lost VELs closely matched the known binding motif for ELF3, a member of the ETS family that is a known mediator of vascular inflammation. For the two *de novo* motifs found among the*Apc*^*Min*/+^ lost VELs, one matches the binding motif for ELF3, and another matches a known motif that is part of the IFN-stimulated response element (ISRE). The ISRE is a binding site for interferon regulatory factors (IRF), which regulate interferon transcription.

## DISCUSSION

### Dysregulated lipid metabolism in cancer

Previous studies have shown that 15–20 weeks of HFD feeding resulted in multiple epigenetic changes in the intestinal epithelium^[5,6]^. We have previously shown that HFD promotes adenoma development in*Apc*^*Min*/+^ mice as early as three days on HFD^[2]^. Our current study shows that feeding mice an HFD causes epigenetic and transcriptional changes within 3 days in the small intestinal epithelia. Many of these changes are associated with genes involved in lipid metabolism. Our studies suggest that H3K27ac changes play an essential role in mediating the upregulation of many of these genes, including pathways involved in the synthesis, uptake, storage, and oxidation of lipids, all of which have the potential to support accelerated tumor growth^[31–33]^.

Our studies indicate that the transcriptomic upregulation of many of these lipid metabolic genes is mediated by epigenetic changes, as shown by increased H3K27ac. Since fatty acids are essential to meet energy needs and provide the structural building blocks required for cancer cell growth, these metabolic enzymes are often upregulated in cancer^[34,35]^. For example, acetyl-CoA carboxylases may rewire cancer metabolism from glycolysis to lipogenesis to support energy demands for proliferation^[36]^. Stearoyl-CoA desaturases (SCDs) are involved in the synthesis of monounsaturated fatty acids (MUFAs) that are valuable in the formation of cancer stem cells and the promotion of their stem properties^[32]^. MUFAs amplify Wnt signaling via stabilization of β-catenin and LRP5/6 in rodent hepatic stellate cells and tumor-initiating cells^[37]^. SCD1 activity has also been shown to affect Hippo/YAP signaling by promoting the nuclear accumulation of YAP and increasing its transcriptional activity in lung adenocarcinoma cancer stem cells in a Wnt-dependent manner^[38]^. CD36 is commonly upregulated to facilitate increased exogenous fatty acid uptake and has been shown to play a role in tumor cell growth, metastasis, and epithelial-mesenchymal transition in multiple cancers^[31]^. These lipogenic genes are regulated in part by AMPK signaling^[27]^. AMPK signaling is essential in sensing cellular energy levels, promoting the uptake and oxidation of glucose and lipids, and inhibiting anabolic pathways that promote cell growth when energy reserves are low. Although AMPK serves as a tumor suppressor tied to energy regulation in non-cancer cells, it has been shown to act as a tumor promoter in cancer cells by protecting against various stressors, including glucose starvation and extracellular matrix detachment^[39]^. HOMER analysis of the gained VELs also identified a few *de novo* motifs similar to known nucleotide sequences that serve as binding sites for transcription factors involved in lipid metabolism (PPAR-α) and cellular proliferation (Fos, Jun-B).

### Dietary suppression of immune processes

HFD downregulated several genes involved in immune processes. Among these were significant histocompatibility complex (MHC) genes involved in antigen presentation. Although MHC class II expression and function are generally considered restricted to professional antigen-presenting cells, intestinal stem cells (ISCs) located in the epithelium have also been shown to express high levels of MHC class II proteins. They can capture, process, and present antigens to CD4+ T cells^[30]^. Our results are substantiated by recent reports showing that HFD dampens MHC class II expression in murine intestinal epithelial cells, including intestinal stem cells^[30]^. Since MHC-mediated activation of CD4+ T cells is vital for tumor immunity, the loss of such MHC II expression in premalignant ISCs may enhance tumor initiation and contribute to the acceleration of tumor progression by HFD. MHC class II expression was also shown to be mediated by interferon-gamma signaling in the same study, and HFD was shown to downregulate genes involved in this pathway in our study. HOMER analysis of lost VELS also identified a few *de novo* motifs similar to known binding motifs for transcription factors involved in promoting immune processes (IRF, ELF3).

### Role of dietary fat as a tumor promoter

Two specific cancer-promoting pathways were found to be transcriptionally upregulated after three days of HFD. Genes, predominantly ligands and receptors organizing the extracellular matrix, involved in PI3K-Akt signaling were identified to be significantly upregulated in the small intestinal epithelia of WT mice fed HFD compared to those from WT mice fed LFD. In addition, genes coding for glutathione transferases were identified to be significantly upregulated in*Apc*^*Min*/+^ HFD compared to*Apc*^*Min*/+^ LFD samples. These changes are associated with both tumor progression and resistance to chemotherapeutic agents and serve to mitigate the oxidative stress associated with elevated tumor metabolic rates^[27,28]^. Despite these changes, the major tumorigenic pathways, including Wnt, Jak-STAT, MAPK, and mTOR, remained stable after three days of HFD feeding.

While dietary fat is not a tumor initiator since it is not mutagenic, its association with tumor growth suggests its role as a tumor promoter. Given the widespread H3K27ac changes induced by HFD, these results suggest a significant role for epigenetic effects in mediating the acceleration of tumor growth. However, a mutation in one or more key cancer driver genes may be required before supportive metabolic changes promote tumor growth. These observations agree with previous demonstrations that high-fat diets accelerate tumor growth in genetically predisposed tissues, i.e., with mutated oncogene or tumor-suppressed genes^[40,41]^.

### Temporal impact of HFD on the epigenome and transcriptome

When comparing this study with previous studies that fed mice HFD for 15–20 weeks, we identified differing results between the studies that suggest a temporal evolution of the epigenome and transcriptome based on the duration of HFD exposure. Li *et al*. showed that 15–20 weeks of HFD resulted in gained VELs associated with regulation of MAP kinase activity and JAK-STAT cascade, but these prominent cancer pathways were not enriched among gained VELs resulting from three days of HFD in our study^[5]^. Li *et al*. also found lost VELs associated with antigen presentation and processing after 15–20 weeks of HFD, whereas our study did not show these changes after three days of HFD^[5]^. An earlier study found transcriptional downregulation of negative regulators of several pathways involving the EGFR/RTK-RAS-ERK/MAPK cascade, TFG-β signaling, and JAK-STAT signaling; however, these negative regulators were not downregulated after three days of HFD in our study^[6]^. Overall, these time-dependent differences suggest a temporal progression of intestinal epigenomic and transcriptomic changes resembling cancer progression dependent on the duration of HFD exposure.

### Impact of Apc min heterozygosity on epigenomic and transcriptomic changes

Our study showed that diet type had a much more significant impact on epigenomic and transcriptomic variations than the *Apc Min* genotype. Most of the significant processes and pathways identified from analyzing the VELs and DEGs induced by HFD were the same between each genotype. Some VELs and DEGs were identified only when comparing WT HFD and WT LFD, and some were identified only when comparing*Apc*^*Min*/+^ HFD versus*Apc*^*Min*/+^ LFD. However, these differences were not significant enough to be identified when directly comparing WT LFD to*Apc*^*Min*/+^ LFD samples or WT HFD to*Apc*^*Min*/+^ HFD samples. The relatively small differences between the two genotypes suggest that the Wnt signaling pathway is still well-regulated in*Apc*^*Min*/+^ mice relative to WT mice after three days of HFD.

In conclusion, Using ChIP-Seq and RNA-Seq approaches in WT C57 BL6J mice and*Apc*^*Min*/+^ mice. We showed that feeding HFD compared to feeding LFD for only three days resulted in epigenetic and transcriptomic changes in lipid metabolic pathways that provide energy and building blocks to support accelerated tumor growth. HFD was also shown to downregulate immune system pathways, providing a more permissive immunologic environment for tumor progression. Since HFD has been previously shown to accelerate intestinal adenoma development in mice with the *Apc Min* mutation but not in WT mice, these findings indicate the need for genetic predisposition to promote tumor growth^[2]^. Recent studies of early-onset colorectal cancer occurring in individuals younger than age 50 years suggest that obesity and obesogenic diets are contributing factors to the disease^[41]^. Our studies show that short-duration nutritional alterations can modulate epigenetic and transcriptomic factors supporting tumor growth.

## Supplementary Material

Supp. Figures

## Data Availability

The data supporting the manuscript’s findings are available from the corresponding author upon request.
