# Supplementary material for: Epigenetic effects of high-fat diet on intestinal tumorigenesis in C57BL/6J-ApcMin/+ mice
Source: J Transl Genet Genom. Author manuscript; Available in PMC 2023 Feb 17. (PMC9937564; doi:10.20517/jtgg.2022.16)
Supplement: Supp. Figures [file NIHMS1870170-supplement-Supp__Figures.pdf]

Sample ID	Concentration (ng/uL)	RNA Quality Number (RQN)
WT LF 1	63.6	8.5
WT LF 2	56.5	10
WT HF 1	43.5	9
WT HF 2	63.5	9.1
Apc Min/+ LF 1	58.3	9.6
Apc Min/+ LF 2	62.6	9.5
Apc Min/+ HF 1	54.3	9.9
Apc Min/+ HF 2	53.7	10

Supplementary Figure 1. RNA sample isolated from intestinal epithelia used for RNA-SEQ.

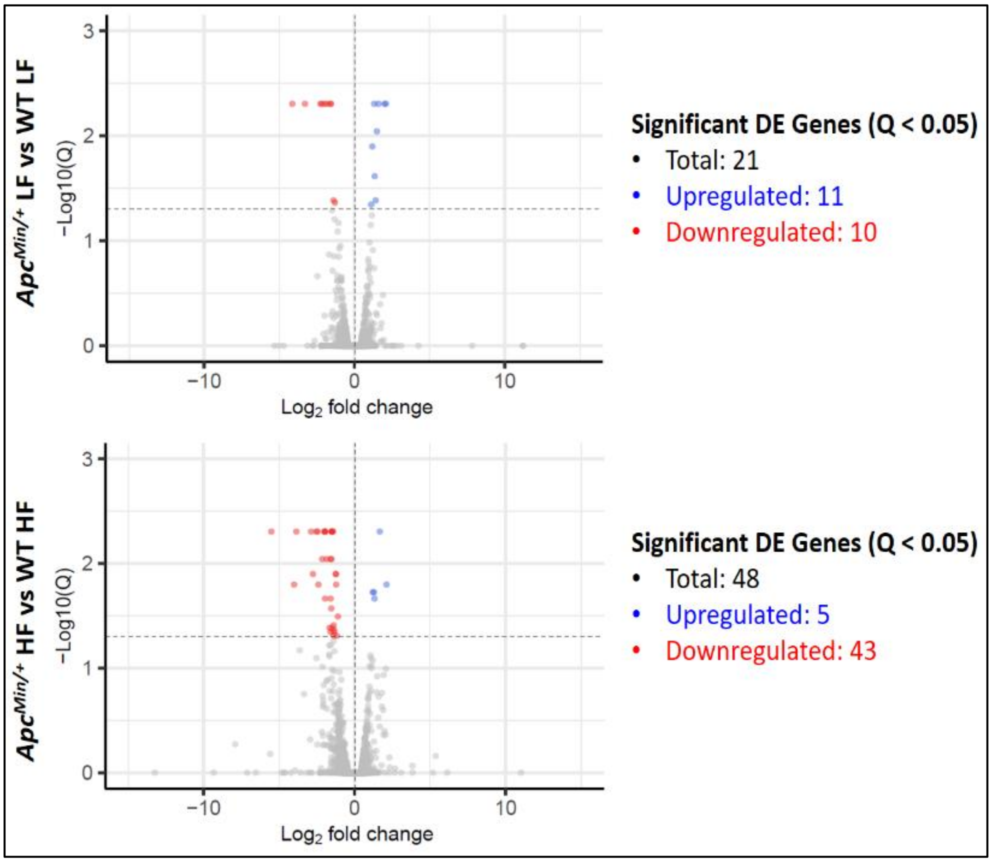

Supplementary Figure 2. Total number of DEGs identified in comparison between WT and *Apc*$^{Min/+}$genotypes (FDR *Q*-value < 0.05)

A

WT HF vs LF			
Gene	VEL Count	log2(AVG VEL FC)	log2(RNA FC)
Cyp4a31	1	1.8251	inf
Glyat	1	1.4384	inf
Scd1	6	2.6448	8.8338
Cyp4a10	4	2.3222	5.7195
Cyp1a1	2	1.2781	4.7616
Acot1	4	2.0103	4.1881
Me1	2	2.8414	4.0958
H2-Q10	1	2.2635	3.8523
Tubb2a	2	0.9836	3.6851
Slc6a3	1	1.5936	3.4256
Mfsd7c	6	1.4547	3.3338
Acacb	2	1.5494	3.2807
Synm	2	1.6349	3.2671
Cntnap1	1	2.6771	3.1936
Tm4sf4	6	1.4684	3.0891
Tcf23	1	1.3154	3.0231
Lamb3	6	1.5426	3.0060
Hmgcs2	9	1.9713	2.9736
Rab30	1	1.1868	2.9706
Slc22a3	2	1.5280	2.9217
Pmm1	1	0.9160	2.7500
Matn2	1	1.5967	2.5532
Dgkh	2	1.1636	2.5289
Acaa1b	1	1.3021	2.4822
Insig1	1	1.3899	2.3838
Scd2	3	1.2276	2.3784
Pdgfrb	1	1.1956	2.3503
Paqr7	1	1.2031	2.1900
Aqp3	1	1.0151	2.1856
Gsta3	1	0.9468	2.1819
Acot2	1	2.0147	2.1155
Acsl3	3	1.1496	2.1145
Utp14b	1	0.8552	2.0892
Slc28a1	1	0.9674	2.0281
Akr1c19	1	0.9547	2.0114
Plscr4	7	1.4981	2.0007
Ugt2b5	1	1.0938	1.9639
Hsd3b3	2	1.1464	1.9482
1190002N15Rik	3	1.4178	1.9481
Mfsd2a	3	1.2612	1.9435
Marc1	1	0.7392	1.9036
Ltc4s	1	0.7311	1.7846
Slc16a12	1	2.5955	1.7815
Pdk4	3	1.5091	1.7584
Retsat	2	1.4744	1.7530
Aldh1a1	4	1.2756	1.7076
Srebf1	2	0.9573	1.6952
Por	4	1.2158	1.6419
Susd2	1	1.0966	1.6325
Cd36	4	2.3184	1.6211
Rgn	2	1.3124	1.6108
Angptl4	1	1.1914	1.5998
Csf1r	4	1.2424	1.5699
Mgst1	1	0.9078	1.5297
Mgll	4	1.7453	1.5204
Celf5	1	0.8286	1.4841
Ces1f	4	1.1727	1.4596
Plin2	3	1.1343	1.4377
Ahcy	3	1.5493	1.4121
Acot4	2	2.0505	1.3997
Nceh1	4	1.4832	1.3834
Aadac	1	1.1431	1.3805
Hsd17b4	2	1.6749	1.3494
Hsdl2	1	1.9612	1.3243
Slc27a2	1	0.9780	1.3239
Gpam	1	1.6149	1.2958
Ccdc114	1	0.9502	1.2766
Acaa2	3	1.3198	1.2438
Ces2b	5	1.4227	1.2275
Mcm2	1	1.8104	1.2104
Mfge8	2	1.0856	1.1879
Gramd4	2	1.2645	1.1395
Pfkl	2	1.4513	1.1133
Aqp7	1	1.0151	1.1093
Vkorc1l1	4	1.9078	1.0824
Scarb2	1	1.2717	1.0617

Apc Min/+ HF vs LF			
Gene	VEL Count	log2(AVG VEL FC)	log2(RNA FC)
Scd1	7	3.1892	8.9463
Cyp4a10	5	1.8109	5.2691
Synm	4	1.6929	4.7891
Acot1	4	1.6440	4.6354
Acacb	1	2.0985	4.6244
Tcf23	1	1.4165	4.5879
H2-Q10	1	2.0132	4.2186
Me1	2	2.2074	4.1061
Akr1b8	1	1.1532	4.0611
Slc22a3	3	1.6563	3.8486
Mfsd7c	5	1.3018	3.4020
Hmgcs2	9	1.7167	3.3538
Acox2	1	0.9961	3.1785
Slc6a3	2	1.8274	3.0833
Acot3	1	1.1385	3.0752
Etv4	1	1.9068	3.0669
Lamb3	5	1.3871	3.0498
Rab30	1	1.2208	3.0112
Cntnap1	1	2.3542	2.9849
Kmo	1	1.0714	2.9828
Thsd4	1	0.9197	2.9568
Spry4	1	1.6754	2.9426
Aldh1a1	2	1.6686	2.9278
Tm4sf4	6	1.6357	2.9060
Bbox1	1	0.8254	2.8582
Akr1c19	1	1.2405	2.8180
Acaa1b	2	1.0928	2.8121
Matn2	1	1.7235	2.7874
Aqp3	2	1.1285	2.6315
Papln	1	0.8785	2.6271
Tubb2a	2	1.0132	2.4580
Plscr4	6	1.7182	2.4540
Acadl	1	1.0463	2.2102
Adh7	2	1.0112	2.1293
Slit2	3	1.4245	2.0662
Rusc2	2	1.6612	2.0556
Mgst1	1	0.9525	2.0370
Pmm1	1	0.7843	2.0050
Rgn	2	1.6999	1.9992
Por	3	1.6582	1.9845
Dgkh	4	1.1095	1.9753
Mgll	4	1.8665	1.9239
Retsat	2	1.3454	1.9165
Ltc4s	1	0.8796	1.9060
Acot2	1	1.7317	1.9012
Mfsd2a	2	1.1770	1.8828
44256	1	0.9332	1.8817
Slc20a1	1	1.0020	1.8744
Dus2	1	0.9354	1.8160
N4bp2l1	1	1.4722	1.8033
Plscr1	3	1.7581	1.7734
Cd36	3	2.0604	1.7717
Mcm2	1	1.7004	1.7503
Nkain1	2	0.9954	1.7489
Aldh3a2	1	2.3561	1.7254
Lipg	2	1.8640	1.7158
Paqr7	1	1.0397	1.6979
Acsl3	2	1.2405	1.6947
Hsd17b4	3	1.5514	1.6913
Slc16a12	1	2.2866	1.6465
Ddah1	2	1.3409	1.6337
Plekho2	1	1.0131	1.6150
Slc25a33	2	1.2735	1.5628
Acta1	1	1.0500	1.5582
Slc5a3	1	1.3764	1.5551
Hsdl2	1	1.9266	1.5539
Aadac	1	0.9440	1.5441
Acot4	2	1.6020	1.5346
Adh1	2	1.0112	1.4979
Ahcy	2	1.6064	1.4937
Gramd4	1	1.7378	1.4933
Scd2	1	1.1045	1.4859
Plin2	2	1.1396	1.4856
Suco	4	1.3605	1.4449
Nceh1	3	1.6517	1.4305
Ccdc114	1	1.1182	1.4294
Hsd17b13	1	0.9545	1.4267
Banp	1	0.9880	1.3810
Phlda1	3	0.9948	1.3583
Ppara	1	1.1587	1.3421
Insig1	1	1.4053	1.3358
Acaa2	3	0.1923	1.3218
Celf5	1	0.7290	1.3193
Shmt2	1	1.4400	1.3031
Fasn	2	1.2183	1.2994
Eci2	2	1.3273	1.2898
Bri3bp	2	1.0297	1.2647
Pdk4	1	1.8697	1.2341
1700037H04Rik	1	1.2749	1.2323
Ephb2	1	1.8145	1.2008
Slco2b1	1	1.0760	1.1950
Vkorc1l1	4	1.5983	1.1628
Pfkl	1	1.0095	1.1495
Thnsl2	3	1.7797	1.1372
Slc25a20	1	0.9735	1.1282
Rhbdd2	3	1.6582	1.1276
Cotl1	2	1.0701	1.1230
Gusb	4	1.5983	1.0839
Def8	2	1.1370	1.0761

B

WT HF vs LF			
Gene	VEL Count	log2(AVG VEL FC)	log2(RNA FC)
Pml	1	-1.0372	-1.0671
Gprin3	1	-1.0630	-1.0817
Fzd7	2	-1.4557	-1.1269
Trim36	1	-1.1265	-1.1296
Elmo1	1	-1.1089	-1.1430
Slc52a3	1	-0.9582	-1.1479
4930402H24Rik	3	-0.9335	-1.2438
Ppargc1a	1	-0.8366	-1.2632
Ifit1	1	-0.9511	-1.3006
Rnf186	2	-1.5875	-1.3061
Aplp1	1	-1.0261	-1.3517
Psmb8	1	-0.8169	-1.4555
Ciita	1	-1.6888	-1.4635
Trim30a	1	-1.0093	-1.4665
Smpdl3b	1	-1.0960	-1.6654
Mtus2	1	-1.0355	-1.6786
Cpne2	2	-1.5168	-1.6947
Syde1	1	-0.8098	-1.6958
Neurl1b	1	-0.8511	-1.7081
Ifit1bl1	2	-0.9552	-1.7130
Oas3	1	-1.1605	-1.7143
Bmf	1	-1.0047	-1.7477
Ift22	2	-1.4320	-1.7672
Upp1	3	-1.1952	-1.7674
Slfn5	1	-1.5570	-1.8966
Socs3	1	-1.5218	-1.9714
Casp4	1	-1.0714	-2.0342
Herc6	1	-0.9801	-2.0394
Tifa	1	-1.2468	-2.1397
Npl	2	-1.1851	-2.3182
Alpk1	1	-1.4762	-2.3828
B3galt5	2	-1.5709	-2.4540
Aldh4a1	2	-1.3513	-2.4660
Gm5431	2	-1.1705	-2.6197
Bco1	2	-1.5132	-2.7558
Slfn2	1	-1.0332	-3.0138
Barx2	4	-1.6059	-3.4121
Nos2	1	-1.9492	-3.7120
Ubd	1	-2.1216	-4.3912

Apc Min/+ HF vs LF			
Gene	VEL Count	log2(AVG VEL FC)	log2(RNA FC)
Trim12c	1	-1.0005	-1.1353
Dio1	2	-0.9621	-1.2214
Slc52a3	1	-1.2808	-1.2678
Camk2n1	1	-1.1773	-1.2690
Akr1b7	1	-1.0261	-1.2758
Slc9a3	1	-0.8392	-1.3062
Aplp1	1	-0.9051	-1.3438
Foxq1	1	-1.2632	-1.4454
Ift22	1	-1.4658	-1.5382
Cpne2	1	-1.0225	-1.6347
Setbp1	1	-0.8823	-1.6502
Spats2l	2	-1.1516	-1.6772
Arntl	1	-1.0672	-1.7292
Apol9b	1	-1.0097	-1.7670
Trim12a	1	-1.0005	-1.8376
Dock3	1	-1.0091	-1.8429
Upp1	3	-1.2993	-1.8682
Lama3	1	-0.8955	-1.8807
Pdx1	2	-1.6288	-1.9527
Tnfsf13b	2	-1.1127	-1.9999
Mtus2	1	-1.1643	-2.0845
Fgf15	1	-1.1306	-2.3203
Ifi47	1	-1.1530	-2.3206
Trim30a	1	-0.9541	-2.3402
Ifit1	1	-1.3156	-2.4215
Igtp	1	-1.0078	-2.4431
Fbxo32	1	-1.1646	-2.4660
Car2	1	-1.7294	-2.4669
Apol10a	1	-1.3385	-2.4933
Fam189a2	1	-1.2998	-2.5927
Herc6	1	-1.0173	-2.8083
AA467197	1	-1.4844	-2.8981
Slfn5	1	-1.7473	-2.9005
Slc40a1	2	-1.3528	-3.0115
S100g	1	-1.2686	-3.1431
Akp3	1	-0.9669	-3.1750
Gm5431	2	-1.5081	-3.2775
Trpv6	2	-1.6601	-3.5628
Ifit1bl1	2	-1.3009	-3.6906
Bco1	2	-1.2991	-4.4173

Supplementary Figure 3. (A) Upregulated genes predicted to be associated with gained H3K27ac and (B) downregulated genes predicted to be associated with lost H3K27ac were identified when comparing HFD and LFD for both genotypes.

***WT* HF vs LF**

Gene	Chromosome	Log2(FC)
AF366264	8	0.957246
Axin2	11	1.262206
Camk2g	14	1.27222
Ccnd1	7	0.962548
Ctbp2	7	0.897299
		1.951858
Dkk4	8	1.224642
Fzd2	11	1.549727
Jun	4	1.438616
Lrp5	19	1.183137
Lrp6	6	1.092265
Myc	15	1.281227
Nfatc1	18	1.381062
Nfatc2	2	1.374678
Nfatc3	8	0.870843
Plcb1	2	1.808879
Plcb4	2	1.808879
Rbx1	15	1.926345
Ror2	13	1.20083
		1.091875
		1.032132
Rspo1	4	1.216613
Ryk	9	1.191162
Siah1a	8	0.731683
		0.730792
		0.681032
Tbl1xr1	3	1.549353
		1.341887
		0.949417
Wnt3	11	1.121668
Wnt8b	19	3.367555
		2.365746
		2.329874
		2.319161
Camk2a	18	-1.43404
Cul1	6	-1.06219
Fzd1	5	-1.30584
Fzd7	1	-1.09343
		-1.94061
Mapk10	5	-1.0089
Ruvbl1	6	-1.05596

***Apc Min* HF vs LF**

Gene	Chromosome	Log2(FC)
AF366264	8	1.130094
Axin2	11	1.973693
Camk2b	11	0.993982
Camk2g	14	1.64773
Ctbp2	7	2.200558
Ctbp3	7	1.541006
Ctbp4	7	0.788489
Ctnnb1	9	1.378863
Dkk4	8	1.440516
Fzd2	11	1.557902
Jul	4	1.224063
Jun	4	1.540921
Lrp5	19	0.900883
Myc	15	1.229607
Nfatc1	18	1.212435
Nfatc1	18	1.424671
Nfatc2	2	1.230217
Nfatc3	8	0.935368
Plcb1	2	2.586879
Plcb2	2	1.14806
Plcb4	2	2.586879
Ror1	4	1.413505
Ror2	13	1.426124
		0.984884
Rspo1	4	1.835295
		1.376297
Siah1a	8	0.876798
Tbl1xr1	3	1.344234
Tbl1xr2	3	1.071013
Tcf7l2	19	0.83646
Wif1	10	0.911754
Wnt3	11	0.913675
Wnt7b	15	1.158716
Wnt8b	19	3.945001
		2.581491
		2.897042
		3.471081
Wnt9b	11	0.913675
Crebbp	16	-0.94913
Fzd7	1	-1.21369
Map3k7	4	-1.40045
Ppard	17	-0.82339
Tbl1xr3	3	-1.29325

Supplementary Figure 4. Identified VELs predicted to be associated with genes involved in the KEGG Pathway "Wnt Signaling Pathway"

WT HF vs LF Gained VELs

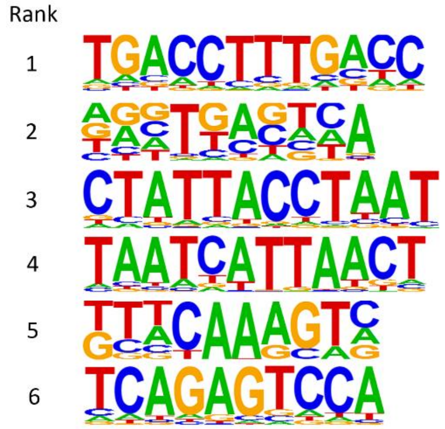

Rank	P-value	% of Targets	% of Backgrounds	Best Match/Details
1	1.00E-89	59.69%	32.43%	Nr2f6/MA0677.1/Jaspar(0.971)
2	1.00E-18	29.27%	18.91%	Fos(bZIP)/TSC-Fos-ChIP-Seq(GSE110950)/Homer(0.877)
3	1.00E-16	10.57%	4.89%	TEF/MA0843.1/Jaspar(0.746)
4	1.00E-15	15.25%	8.48%	HNF1b(Homeobox)/PDAC-HNF1B-ChIP-Seq(GSE64557)/Homer(0.893)
5	1.00E-13	34.41%	25.23%	HNF4G/MA0484.2/Jaspar(0.791)
6	1.00E-12	33.87%	24.99%	HNF4G/MA0484.2/Jaspar(0.827)

Apc$^{Min/+}$ HF vs LF Gained VELs

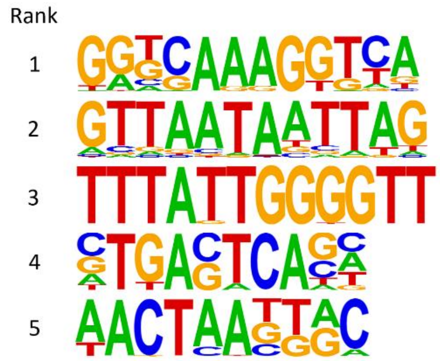

Rank	P-value	% of Targets	% of Backgrounds	Best Match/Details
1	1.00E-97	36.78%	11.71%	PPARa(NR),DR1/Liver-Ppara-ChIP-Seq(GSE47954)/Homer(0.953)
2	1.00E-17	9.76%	3.75%	HNF1b(Homeobox)/PDAC-HNF1B-ChIP-Seq(GSE64557)/Homer(0.820)
3	1.00E-14	1.71%	0.12%	HOXA13/MA0650.2/Jaspar(0.773)
4	1.00E-14	36.11%	25.41%	JunB(bZIP)/DendriticCells-Junb-ChIP-Seq(GSE36099)/Homer(0.957)
5	1.00E-12	6.82%	2.65%	HOXD3/MA0912.2/Jaspar(0.769)

WT HF vs LF Lost VELs

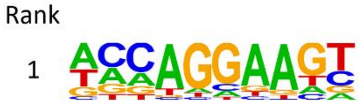

Rank	P-value	% of Targets	% of Backgrounds	Best Match/Details
1	1.00E-13	48.70%	25.82%	ELF3(ETS)/PDAC-ELF3-ChIP-Seq(GSE64557)/Homer(0.958)

Apc$^{Min/+}$ HF vs LF Gained VELs

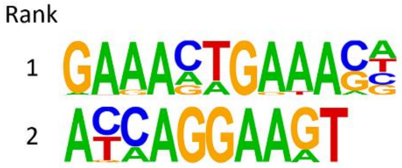

Rank	P-value	% of Targets	% of Backgrounds	Best Match/Details
1	1.00E-28	17.03%	2.92%	ISRE(IRF)/ThioMac-LPS-Expression(GSE23622)/Homer(0.960)
2	1.00E-25	36.76%	14.55%	ELF3(ETS)/PDAC-ELF3-ChIP-Seq(GSE64557)/Homer(0.960)

Supplementary Figure 5. Notable *de novo* motifs were identified from the VELs using HOMER analysis. The percentage of target sequences as well as the percentage of background sequences containing the motif was noted. Known motif that most closely matches the *de novo* motifs are also noted.

WT VELs				
Locus	Log2(Fold-Change)	Q-value	-Log(Q)	Genes
chr9: 86593210-086594683	3.446945748	4.50E-21	20.35	Me1 (-4000)
chr19: 44489642-44492211	3.367555271	1.70E-23	22.77	Wnt8b (-77035), Scd1 (-8728)
chr19: 44480332-44483612	3.340702254	5.72E-29	28.24	Scd1 (+227)
chr4: 115176360-115178539	2.909126154	1.55E-11	10.81	Cyp4a10 (-13419), Cyp4a14 (-8703)
chr5: 17382694-17383654	2.776266782	5.27E-11	10.28	Gnat3 (-85265), Cd36 (-27564)
chr5: 17385604-17387914	2.645476921	1.25E-13	12.90	Gnat3 (-81680), Cd36 (-31149)
chr19: 44501866-44503173	2.365745908	1.72E-05	4.76	Wnt8b (-65442), Scd1 (-20321)
chr4: 115185859-115188695	2.263902491	5.01E-17	16.30	Cyp4a10 (-3592)
chr5: 17315839-17317427	2.073964175	0.00014189	3.85	Cd36 (+38977), Sema3c (+236534)
chr4: 115190229-115192416	2.051807598	3.35E-09	8.48	Cyp4a10 (+454)
chr5: 114592757-114596921	1.999563769	7.38E-19	18.13	Acacb (-1705)
chr4: 94733979-94736057	1.438615901	7.63E-06	5.12	Fggy (-489180), Jun (-16105)
chr19: 44367741-44370983	1.306706028	0.000665067	3.18	Scd4 (-38220), Scd2 (+1196)
chr15: 61728278-61730622	1.281227212	0.008045716	2.09	A1bg (-976625), Myc (-87496)
chr11: 108816516-108818471	1.262205576	0.019059714	1.72	Axin2 (+35831), E030025P04Rik (+188189)
Apc Min/+ **VELs**				
Locus	Log2(Fold-Change)	Q-value	-Log(Q)	Genes
chr19: 44480332-44483612	4.136092828	1.68E-41	40.78	Scd1 (+227)
chr19: 44489642-44492211	3.945001263	1.21E-31	30.92	Wnt8b (-77035), Scd1 (-8728)
chr19: 44504425-44506311	3.471080966	4.20E-15	14.38	Wnt8b (-62594), Scd1 (-23169)
chr19: 44501866-44503173	2.897042095	3.93E-08	7.41	Wnt8b (-65442), Scd1 (-20321)
chr9: 86593210-86594683	2.549166354	4.34E-12	11.36	Me1 (-4000)
chr5: 17382694-17383654	2.309785836	1.10E-07	6.96	Gnat3 (-85265), Cd36 (-27564)
chr4: 115225498-115225939	2.266004995	0.003806215	2.42	Cyp4a31 (-10584), Cyp4a10 (+34850)
chr5: 114592757-114596921	2.098515026	1.28E-20	19.89	Acacb (-1705)
chr11: 108816516-108818471	1.973692953	1.42E-05	4.85	Axin2 (+35831), E030025P04Rik (+188189)
chr4: 115185859-115188695	1.79929721	1.85E-10	9.73	Cyp4a10 (-3592)
chr4: 94733979-94736057	1.540920907	1.12E-06	5.95	Fggy (-489180), Jun (-16105)
chr11: 120691508-120696082	1.261634714	0.001850552	2.73	Fasn (-8303), Ccdc57 (+100391)
chr15: 61728278-61730622	1.229606782	0.014500981	1.84	A1bg (-976625), Myc (-87496)
chr4: 94742053-94743449	1.224062698	0.029967415	1.52	Fggy (-481447), Jun (-23838)
chr19: 44340446-44342794	1.104469296	0.004176435	2.38	Scd2 (-26546), Scd3 (+63858)

Supplementary Figure 6. Top fifteen significant VELs with the highest H3K27ac activity fold-change between HFD and LFD for WT and *Apc*$^{Min/+}$ mice. Genes located near the VELs were identified with their relative distance shown.

Gene	Locus	Log2(Fold-Change)	Q-value	-Log(Q)
**WT DEGs**				
**Gene**	**Locus**	**Log2(Fold-Change)**	**Q-value**	**-Log(Q)**
Scd1	chr19: 44468939-44482199	8.8338	0.004937	2.306511
Cyp4a10	chr4: 115190891-115206254	5.7195	0.004937	2.306511
Me1	chr9: 86474969-86595286	4.09577	0.004937	2.306511
Acacb	chr5: 114615526-114700767	3.2807	0.004937	2.306511
Lamb3	chr1: 195128187-195170072	3.00596	0.004937	2.306511
Pdgfra	chr5: 75548315-75594229	2.51629	0.004937	2.306511
Pdgfrb	chr18: 61204803-61244721	2.35033	0.004937	2.306511
Gsta3	chr1: 21230665-21255656	2.18186	0.004937	2.306511
Col6a2	chr10: 76058500-76086149	1.99458	0.036739	1.43487
Cd36	chr5: 17287507-17394777	1.62109	0.004937	2.306511
Mgst1	chr6: 138089057-138105273	1.52966	0.004937	2.306511
H2-T23	chr17: 36131448-36175119	-1.21923	0.02162	1.665152
H2-DMa	chr17: 34259776-34276046	-2.18605	0.004937	2.306511
H2-Ab1	chr17: 34400171-34406363	-2.3784	0.004937	2.306511
H2-DMb1	chr17: 34290135-34297174	-2.82595	0.012603	1.899512
***Apc Min/+* DEGs**				
**Gene**	**Locus**	**Log2(Fold-Change)**	**Q-value**	**-Log(Q)**
Scd1	chr19:44468939-44482199	8.94627	0.004937	2.306511
Cyp4a10	chr4:115190891-115206254	5.26914	0.004937	2.306511
Acacb	chr5:114615526-114700767	4.62441	0.004937	2.306511
Me1	chr9:86474969-86595286	4.10613	0.004937	2.306511
Lamb3	chr1:195128187-195170072	3.04979	0.004937	2.306511
Col1a2	chr6:4455696-4491543	2.18197	0.004937	2.306511
Col1a1	chr11:94797583-94813170	2.08095	0.004937	2.306511
Mgst1	chr6:138089057-138105273	2.03698	0.004937	2.306511
Pdgfra	chr5:75548315-75594229	1.84919	0.04712	1.326797
Cd36	chr5:17287507-17394777	1.77168	0.004937	2.306511
Gstm3	chr3:107766613-107772092	1.60158	0.004937	2.306511
H2-DMa	chr17:34259776-34276046	-2.31638	0.004937	2.306511
H2-Aa	chr17:34419695-34424716	-3.04097	0.004937	2.306511
H2-Ab1	chr17:34400171-34406363	-3.32936	0.004937	2.306511
H2-DMb1	chr17:34290135-34297174	-4.14059	0.004937	2.306511

Supplementary Figure 7. Notable differentially expressed genes identified between HFD and LFD for WT and *Apc*$^{Min/+}$ mice.
